# Lactobacillus johnsonii BS15 combined with abdominal massage on intestinal permeability in rats with nonalcoholic fatty liver and cell biofilm repair

**DOI:** 10.1080/21655979.2021.1954134

**Published:** 2021-09-13

**Authors:** Wei Zhang, Huanan Li, Na Zhao, Xiongfei Luo, Siwen Liu, an Bao, Yingying Chen, Haiteng Wang, Junshi Wang, Jingui Wang

**Affiliations:** a Department of Massage, The First Affiliated Hospital of Tianjin University of Chinese Medicine, Tianjin, China; b National Clinical Research Center for Chinese Medicine Acupuncture and Moxibustion, Tianjin, China

**Keywords:** nonalcoholic fatty liver disease, lactobacillus, abdominal massage, biochemical indexes of liver tissue

## Abstract

This study aimed to analyze the effect of lactobacillus johnsonii BS15 (isolation of homemade yogurt from Ahu Hongyuan Grassland) combined with abdominal massage on intestinal permeability in rats with nonalcoholic fatty liver disease (NAFLD) and cell biofilm repair. Forty-five rats were divided randomly into five groups, four of which were fed with high-fat diet to establish NAFLD models. According to the treatment methods, they were grouped into group A (lactic acid bacteria feeding), group B (abdominal massage), group A + B (a combination of the two methods), model group (distilled water feeding), and normal group (distilled water feeding). Then, the pathological indexes of liver and intestinal permeability were observed. FITC-Dextran content of the model group elevated markedly compared with normal group (*P* < 0.01), indicating that the intestinal permeability of NAFLD rats fed with high-fat diet increased. The intestinal permeability of groups A, B, and A + B was lower sharply than that of model group (*P* < 0.01), and the effect of group A + B was the most obvious. HE staining of liver tissues showed that combined treatment could improve structural changes in liver cells caused by modeling and restore the normal structure of intestinal cells. Lactobacillus combined with abdominal massage was better than two treatments alone, further promoting the permeability of intestinal mucosa in NAFLD rats and repair biofilm of hepatocytes. The results initially verified the intervention effect of abdominal massage on intestinal mucosal permeability, and further revealed the mechanism of abdominal massage in treatment of NAFLD by improving intestinal mucosal barrier permeability.

## Graphical abstract

**Figure:**
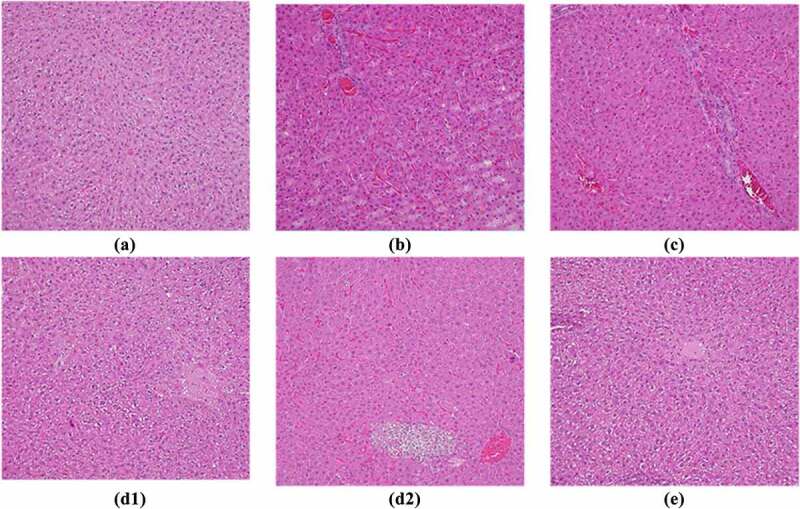

## 1. Introduction

Nonalcoholic fatty liver disease (NAFLD) is a common chronic metabolic liver disease, and its clinical features include no history of excessive drinking, and free fatty acid (FFA) accumulation in the liver to cause the occurrence of parenchymal fatty degeneration, accumulation, and low-grade inflammation. NAFLD disease spectrum includes simple nonalcoholic fatty liver (NAFL), as well as nonalcoholic steatohepatitis (NASH), liver cirrhosis, and hepatocellular carcinoma derived from it [1]. According to research, people believe that the prognosis of NAFL in the NAFLD disease spectrum is good and curable; 20–30% of NAFLD patients will develop NASH, and chronic inflammation in the liver will induce fibrosis. At this time, the disease will continue to progress irreversibly. About 60% of NASH patients will further develop liver cirrhosis and portal hypertension, and even worse, subacute liver failure and NAFLD-related liver cancer diseases will eventually occur [2]. NAFLD occurs in all age groups, of which adults account for 30%, and this proportion is rising rapidly with the prevalence of obesity, type 2 diabetes, and metabolic syndrome [3].

In recent years, the mechanism of NAFLD has been researched a lot [4,5]. The currently known ones include oxidative stress, inflammation, changes in the intestinal environment, and insulin resistance [6]. The liver and intestines share the same embryonic origin, and there are many internal connections between various anatomical and biological functions, and they share the same functional units, such as blood and nutrition. Therefore, the research on the intestinal tract has gradually become a focus of attention after Marshall (1998) [7] formally proposed the concept of ‘enteric-hepatic axis.’ Intestinal mucosal permeability is a critical index of the intestinal mucosal barrier function. The relationship between intestinal mucosa and hepatic steatosis has been initially confirmed in studies on the mechanism of alcoholic fatty liver disease [8]. The imbalance of intestinal flora may change intestinal permeability, so as to create a favorable microenvironment for bacterial overgrowth, mucosal inflammation, and transfer of invasive pathogens and harmful by-products, thus affecting liver fat formation and accelerating the process of pro-inflammatory and fibrosis [9]. There are not many treatments for NAFLD, which has prompted a variety of potentially therapeutically valuable drugs to be used to explore its therapeutic effects on NAFLD. Since most NAFLD patients are overweight and obese and lactobacillus has anti-obesity function, lactobacillus is considered to be a new strategy for the treatment of NAFLD [10,11]. Lactobacillus, a member of lactic acid bacteria, is one of the normal flora of the human intestinal tract and plays a critical role in maintaining intestinal microecological stability [12,13]. Previous studies have pointed out that lactobacillus has a good therapeutic effect on NAFLD. Moreover, Chinese medicine has been extensively applied in the treatment of NAFLD in recent years. Doctors of traditional Chinese medicine believe that NAFLD is formed due to damp heat combined with phlegm-fatigue, and the liver veins obstructed by arthralgia. Therefore, it is very important to soothe the liver and invigorate the spleen, and promote blood circulation and fatigue. Besides, the treatment of NAFLD patients has been initiated with targeted abdominal massage and special acupuncture point pressing. The abdominal massage can drive the movement of abdominal muscles, increase the permeability of liver cells, further improve the microcirculation disorder, help promote the consumption and transport of fat in the liver and reduce its accumulation in the liver, so as to achieve the NAFLD treatment [14,15].

Up to now, there have been many studies on the drug treatment of NAFLD, and many new drugs have entered the clinical trial stage, but their safety and effectiveness need to be further tested, which has prompted more research on the exploration of NAFLD treatment methods. Recent clinical studies have shown that lactobacillus and abdominal massage have good clinical effects on NAFLD, but there is no report on the research of combining the two in the treatment of NAFLD. In this experiment, it was hoped that the rats were induced to establish the NAFLD models through high-fat diet, and lactobacillus combined with abdominal massage was used for intervention treatment. Then, it was compared with lactobacillus or abdominal massage alone to observe the effects of combined treatment on liver histomorphology, liver function, and intestinal mucosal permeability of SD rats, so as to contribute new ideas to the prevention and treatment of NAFLD.

## 2. Materials and methods

### 2.1 Materials and reagents

Forty-five healthy male Sprague-Dawley (SD) rats were 4–5 weeks old, weighing between 120 and 140 g, which were provided by the Experimental Animal Center of Tianjin University of Traditional Chinese Medicine with a laboratory animal certificate (No. 81,503,671). The rats were reared under the conditions of specific pathogen-free (SPF), and there were 12 hours of artificial light and 12 hours of darkness. What is more, the rearing environment was 22–26°C, relative humidity was 50–60%, and adaptive feeding was for 1 week. In addition, the operation of this experiment was in line with the relevant regulations of the *Experimental Animal Ethics Certificate*.

Lactobacillus johnsonii BS15 was applied in this experiment, which was isolated from the homemade yogurt of Ahu Red Plain Grassland, and the preservation number of the strain was CCTCC NO: M2013663.

### 2.2 Construction of NAFLD rat models

After 1 week of adaptive feeding, nine rats were set aside as the control group, and the rats were gavage with normal feed, and the rest of the rats were gavage with high-sugar and high-fat feed every day to construct NAFLD rat models. The high-fat feed formula included 10% lard, 2% cholesterol, 5% sucrose, 0.5% pig bile salt, and 82.5% basic feed.

### 2.3 Grouping and treatment

After 1 week of adaptive feeding, the rats were rolled randomly into the lactobacillus group (group A), an abdominal massage group (group B), a lactobacillus combined with abdominal massage group (group A + B), and a model group based on the random number table method, with nine in each group. Besides, another nine rats were selected as the control group. Except for the control group, other rats were induced to form the NAFLD rat models with high-fat diets. The rats from group A were given with lactobacillus by gavage every day, group B underwent the massage intervention every day, group A + B received the lactobacillus plus abdominal massage intervention every day, the model group and control group were given with an equal volume of distilled water.

The introduction to the operation of abdominal massage in SD rats was as follows. First, the limbs of the experimental rat were tied on the experimental table, making it lie on its back. Then, the right index finger and middle finger were put together, which were buckled on the rat’s abdomen. It was noted that the knuckles of the right palm were slightly arched and the wrist was slightly bent. Afterward, the radial side of the index finger, the surface of the index and middle fingers, and the ulnar side of the middle finger were contacted with the abdomen in turn through the massage method of the wrist joint turning back and forth to complete a rubbing. This operation was repeated for 10 minutes, and the frequency of rubbing should be slow and moderate, namely 20–30 times/min on average. What is more, there was continuous treatment for 28 days. The rats from the model group were also tied to the experimental table with a supine position every day, without any intervention. After 10 minutes, the restraints were untied. In addition, the control group did not receive any intervention [16–18].

### 2.4 Specimen collection

The experimental rats from each group were fasted for 12 hours but could drink water. They were comatose by intraperitoneal injection of 1% sodium pentobarbital, and then, the ileum tissue was found, washed, and absorbed water. Two rats from each group were taken out and their intestinal mucosal tissues were scraped from partial ileum. Next, the tissues were smeared on a glass slice, which was fixed with 4°C and 4% paraformaldehyde. Then, the remaining part of the intestinal mucosa of the ileum was collected, put into a 1.5 mL microcentrifuge tube (Eppendorf (EP) tube), and stored in a refrigerator at −80°C for later use. Afterward, the rats were sacrificed, the liver was quickly separated, the left lateral lobe was taken out, and the blood stains were washed with phosphate buffer saline (PBS) for biochemical testing. Besides, the middle lobe of the liver was cut into 10 mm × 10 mm tissue and placed in 4% paraformaldehyde solution for fixation, which was used for pathological observation.

### 2.5 Evaluation indexes and detection methods

#### 2.5.1 Biochemical indexes of liver tissue

The biochemical indexes that needed to be detected in liver tissue in this experiment mainly included the contents of triglycerides (TG), total cholesterol (TC), alanine aminotransferase (ALT), aspartate aminotransferase (AST), high-density lipoprotein-cholesterol (HDL-C), low-density lipoprotein-cholesterol (LDL-C), and FFA. In this experiment, the used detection kits were all products of Tianjin Institute of Biological Engineering, China. Besides, the detection process of the above biochemical indexes was carried out according to the instructions of the kits.

#### 2.5.2 FITC-Dextran tracer method detection

The FITC-Dextran solution with a pH of 7.2 and a concentration of 20 mg/mL was pre-prepared with 0.1 mmol/L PBS. Before the end of the experiment, the rats were put to death, the abdomen was opened under anesthesia, and the intestine was gently turned over and washed with PBS 3 times. A silk thread (No.3–0) was applied to ligate the end of the ileum, and the intestinal cavity was injected with 0.5 mL of FITC-Dextran solution. Then, the end of the ascending colon was ligated with silk thread (No.3–0). During the operation, the mesenteric blood vessels should not be damaged, and the leakage of FITC-Dextran fluid should be avoided. The intestinal tract was placed in 10 mL of 0.9% sodium chloride solution in a 37°C water bath for 60 minutes. A fluorescence spectrophotometer was employed to detect the fluorescence value, and the content of FITC-Dextran leaked from the intestine was adopted to indicate the change of intestinal permeability.

#### 2.5.3 HE staining of liver tissues

The obtained liver tissue mass was gradually dehydrated with ethanol from a low concentration to a high concentration, and then, placed in xylene. After the tissue block became transparent, it was put in the melted paraffin. The paraffin was completely immersed in the tissue block and embedded, and then, the embedded paraffin block was cut into 5–8 μm slices with a microtome. Then, xylene was employed to remove the paraffin from the slices, and the slices were rinsed with high-concentration ethanol, low-concentration ethanol, and distilled water. Afterward, the HE staining method was adopted to stain the above slices. Each stained slice was dehydrated by absolute ethanol, transparent through xylene, dripped with gum, sealed with a cover glass, and finally, observed under a light microscope.

### 2.6 Statistical methods

The data processing of this study was analyzed by SPSS19.0 version statistical software, the measurement data were expressed as the mean ± standard deviation ($\bar{X}$ ± s), and the count data were represented by the percentage (%). Furthermore, *P* < 0.05 meant that the difference was statistically substantial.

## 3. Results

In this study, there was the main discussion on the effects of Lactobacillus combined with abdominal massage on intestinal permeability and cell biofilm repair in rats with NAFLD. This experiment hoped to induce rats to establish a NAFLD model through a high-fat diet, and lactic acid bacteria with abdominal massage was used for intervention treatment. Besides, it was compared with simple lactic acid bacteria or abdominal massage, to observe the effect of combined treatment on liver tissue morphology, liver function, and intestinal mucosal permeability of SD rats, thereby providing new ideas for the prevention and treatment of NAFLD.

### 3.1 Liver tissue biochemical index results

**Figure 1.**
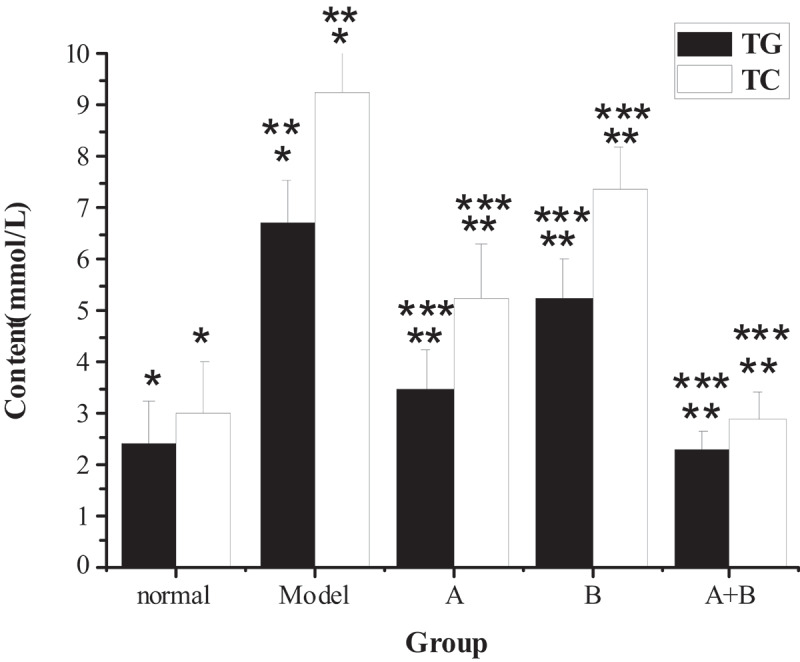
The contents of TG and TC in liver tissues of rats in each group

**Figure 2.**
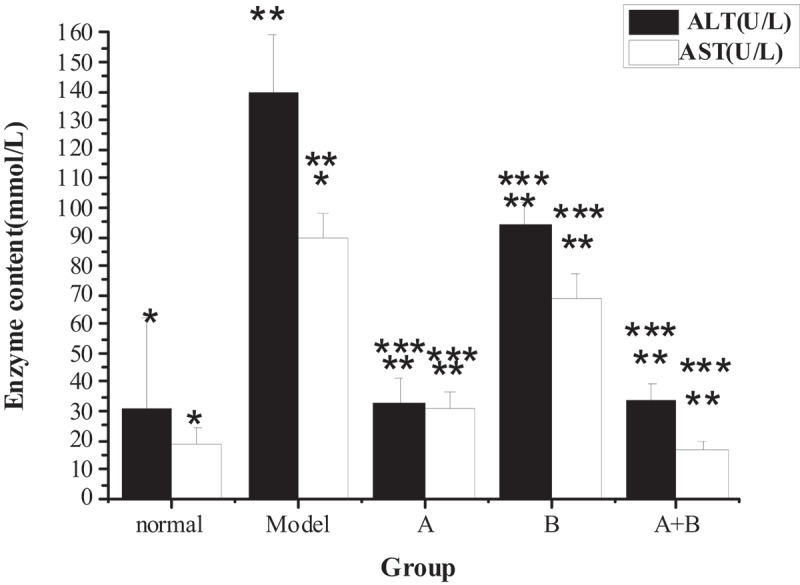
The contents of ALT and AST in liver tissues of rats in each group

**Figure 3.**
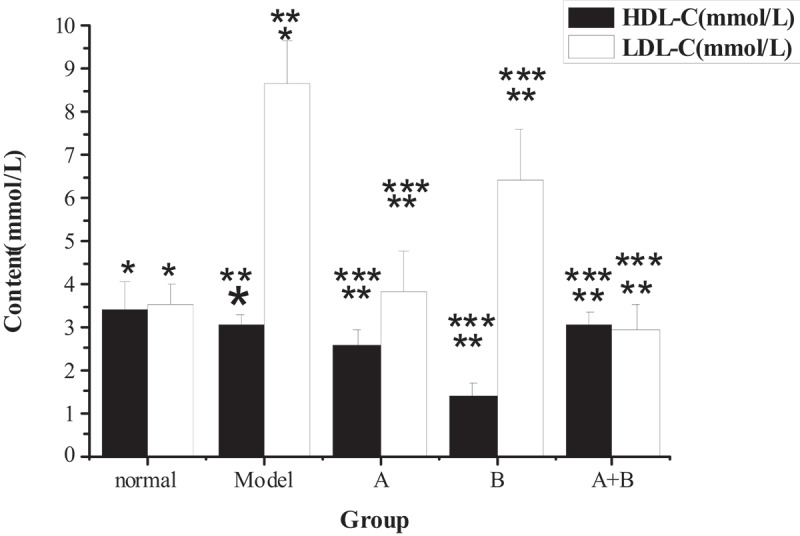
The contents of HDL-C and LDL-C in liver tissues of rats from the 5 groups

**Figure 4.**
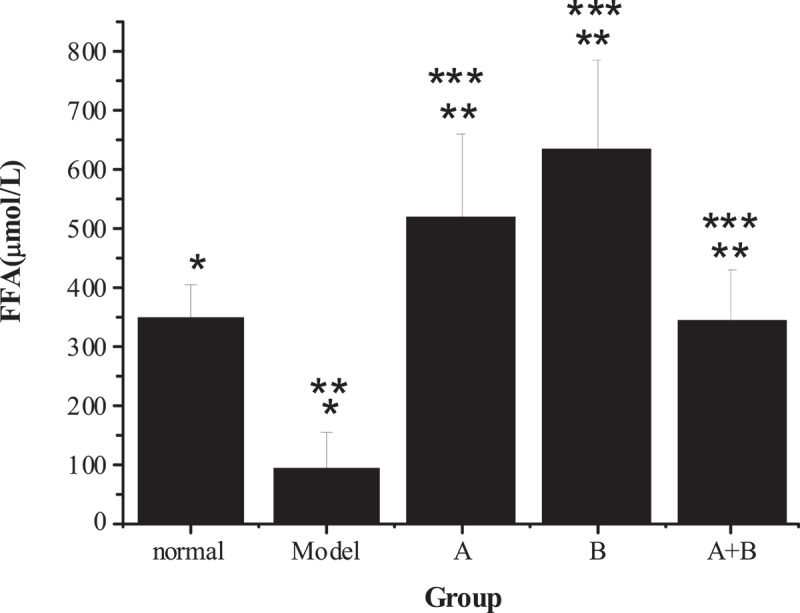
The content of FFA in liver tissues of rats from the 5 groups

The test results of TG, TC, ALT, AST, HDL-C, LDL-C, and FFA in the liver tissues of rats from five different groups were shown in Figures 1–4. The content of TG, TC, ALT, AST, LDL-C, and FFA in the liver of the model group increased hugely in contrast to the content of the control group (*P* < 0.01), but the content of HDL-C reduced sharply (*P* < 0.01). Compared with the model group, the content of TG, TC, ALT, AST, LDL-C, and FFA in rats from group A, group B, and group A + B dropped steeply (*P* < 0.05), while the content of HDL-C rose dramatically (*P* < 0.01). In addition, the difference in each index of group A (except ALT) or group B was statistically remarkable compared with group A + B (*P* < 0.01).

(Note: * indicated that the contents of TG and TC in liver tissue of the model group were significantly increased (*P* < 0.01) compared with the normal group; ** meant that the contents of TG and TC in groups A, B, and A + B were lower than those of the model group (*P* < 0.05); *** showed that the contents of TG and TC in groups A and B were different obviously from those of group A + B (*P* < 0.01).)

(Note: * indicated that the contents of ALT and AST in liver tissue of the model group were increased markedly (*P* < 0.01) compared with the normal group; ** meant that the contents of TG and TC in groups A, B, and A + B were lower than those of the model group (*P* < 0.05); *** showed that the content of AST in groups A and B were different obviously from that of group A + B (*P* < 0.01).)

(Note: * indicated that the content of LDL-C of the model group were increased markedly (*P* < 0.01) compared with the normal group, while the content of HDL-C was decreased sharply; ** meant that the content of LDL-C in groups A, B, and A + B were lower steeply than that of the model group (*P* < 0.05) but the content of HDL-C was higher greatly than that of the model group (*P* < 0.05); *** showed that the contents of LDL-C and HDL-C in groups A and B were different obviously from that of group A + B (*P* < 0.01).)

(Note: * indicated that the content of FFA of the model group was risen markedly (*P* < 0.01) compared with the normal group; ** meant that the content of FFA in groups A, B, and A + B reduced steeply in contrast to that of the model group (*P* < 0.05); *** showed that the content of FFA in groups A and B were different obviously from that of group A + B (*P* < 0.01).)

### 3.2 Fluorescent probe FITC-Dextran tracer method detection results

**Figure 5.**
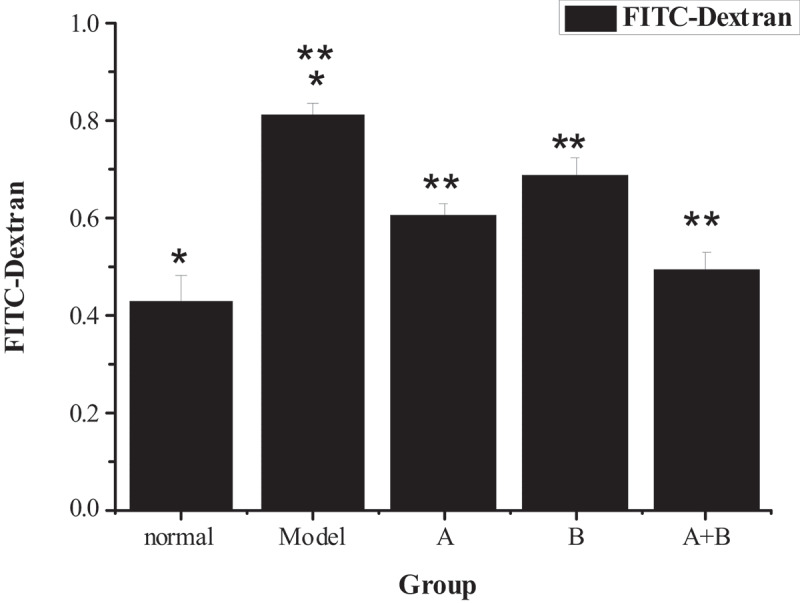
Comparison of FITC-Dextran content of rats from the 5 groups (x ± s, n = 9, and μg/mL)

The detection results of the fluorescent probe FITC-Dextran tracer method are presented in Figure 5. Compared with the control group, the FITC-Dextran content of the model group increased obviously (*P* < 0.01), suggesting that the intestinal permeability of NAFLD rats was improved. However, the FITC-Dextran content of group A, group B, and group A + B was lower sharply than the content of the model group (*P* < 0.01), indicating that lactobacillus treatment, abdominal massage, and lactobacillus treatment combined with abdominal massage could reduce intestinal permeability. Moreover, the combined therapy had the best effect. Through the comparison of the data from the five groups, it revealed the intestinal permeability of rats from the control group < group A + B < group A < group B < the model group.

(Note: * meant that the content of FITC-Dextran in the model group was hugely higher than the content of the normal group (*P* < 0.01); ** showed that the content of FITC-Dextran in groups A, B, and A + B was lower sharply than that of the model group (*P* < 0.01).)

### 3.3 HE staining results of liver tissues

**Figure 6.**
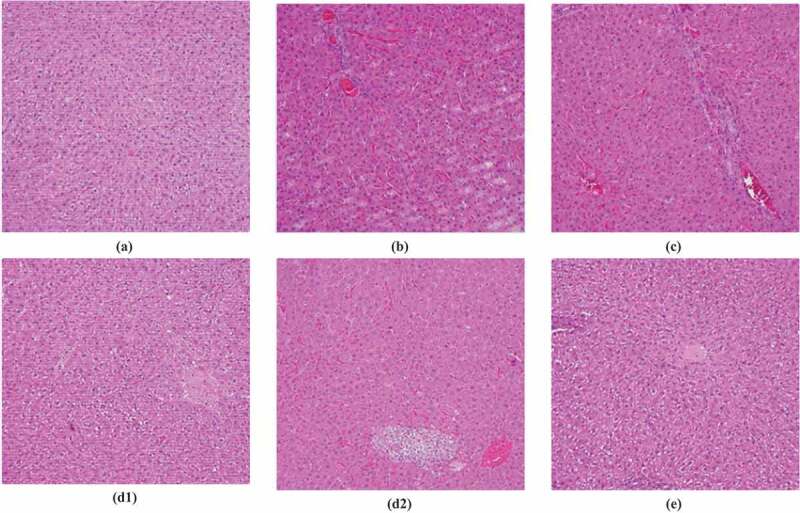
HE staining results of pathological tissue of rats from each group

The results of HE staining of liver tissues showed that the liver cells of rats from the control group were arranged, the structure was intact, and there was no lipid infiltration. In the model group, the liver cells were arranged disorderly, the borders were blurred, and the cytoplasm contained more fatty vacuoles, which proved that the modeling was successful. However, the unclear condition of hepatocytes in group A, group B, and group A + B after treatment was improved, and the number of fatty vacuoles was reduced. Among them, the hepatocytes of group A + B were arranged more closely, and there were no obvious fatty vacuoles, which were closer to the level of the control group. The specific expression of HE staining in the liver pathological tissue of rats from each group is shown in Figure 6.

(Note: A: Normal group; B: Model group; C: Group A; D1: Steatosis staining results of group B; D2: Group B; E: Group A + B.)

### 3.4 Detection results of serum tumor necrosis factor-α, interleukin-1, and interleukin-8

**Figure 7.**
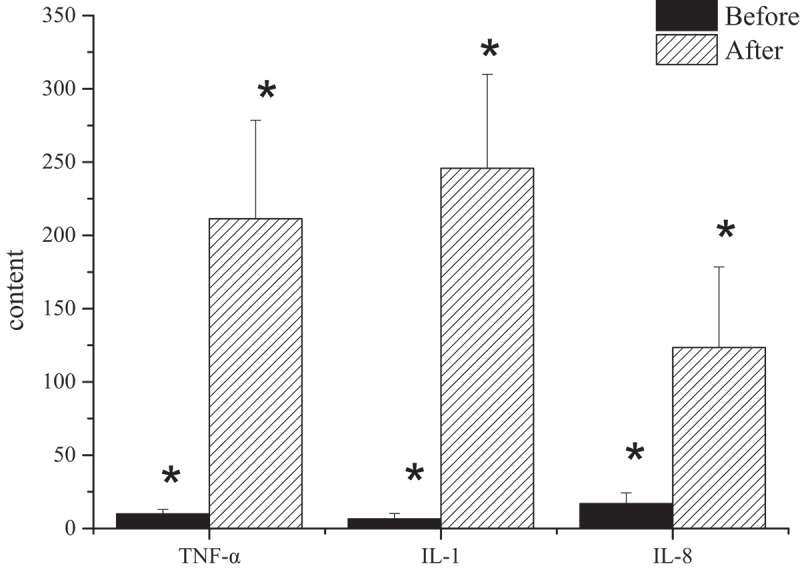
Changes of serum inflammatory cytokines before and after the establishment of the model

In the process of this study, there was a comparison of the changes of serum inflammatory factors in the model group before and after the establishment of the model. The results showed that the contents of serum interleukin-1 (IL-1), IL-8, and tumor necrosis factor-α (TNF-α) in the model group before the establishment of the model dropped hugely compared with those in the model group successfully established with NAFLD, and the comparison was statistically significant (*P* < 0.05) (Figure 7).

(Note: * revealed that the contents of TNF-α, IL-1, and IL-8 in serum of the model group before and after the establishment of the model group were statistically substantial (*P* < 0.05).)

## 4. Discussion

At present, NAFLD is prevalent in the world and has led to adverse effects on national health problems in many countries [19]. Currently known major factors leading to liver steatosis and fibrosis in NAFLD include age, obesity, insulin resistance, type 2 diabetes, and elevated ferritin levels, but the relevant pathological mechanisms resulting in the progression of NAFLD have not yet been resolved. Among them, the ‘second blow’ doctrine has been widely recognized. The theory believes that the ‘second blow’ refers to the blow of lipid peroxygen compounds formed by oxidative stress and lipid peroxidation in liver cells, which can lead to the number and increase in inflammatory factors, such as interleukin-6 (IL-6), IL-18, and tumor necrosis factor-α (TNF-α), to induce inflammation. This hypothesis was similar to the research results of this experiment [4]. Besides, the inflammatory response has an important influence on the occurrence and development of NAFLD. Insulin resistance, oxidative stress, and inflammatory signaling pathways also play a synergistic effect [19–21].

Nowadays, fatty liver caused by hyperlipidemia is often treated with hypolipidemic drugs in clinical practice, but long-term use of the drug will cause side effects such as low liver function and liver lipid metabolism. New and efficient drugs and treatment methods are urgently needed to address the treatment of NAFLD. Recent studies have shown that the occurrence and development of NAFLD are closely related to intestinal flora. The imbalance of the intestinal flora will increase its permeability to bacteria and make harmful substances enter the liver so that autoimmune reactions will damage the liver [6,22–24]. Therefore, improving the intestinal flora and increasing the number of beneficial bacteria can have a certain therapeutic effect on NAFLD. Besides, Tohma et al. (2019) [25] confirmed that Lactobacillus had a good effect on NAFLD. In recent years, NAFLD patients have gradually accepted traditional Chinese medicine treatment. Traditional Chinese medicine classifies ‘fatty liver’ into the categories of diseases, such as ‘phlegm,’ ‘painful abdominal mass,’ and ‘accumulated syndrome,’ believing that fatty liver is caused by liver and spleen disorders. Abdominal massage is one of the most common methods for treating liver and spleen disorders in traditional Chinese medicine, and it has recently been used to treat fatty liver. The core technique of abdomen massage is the ‘rubbing abdomen method,’ which uses direct operation on the patient’s abdomen, and rhythmically applies mechanical force to drive the movement of the abdomen to stimulate the circulation of the liver, spleen, stomach, gallbladder, and other meridians, thereby achieving ‘treating pathogenic accumulation’ and ‘freeing the qi and blood, and maintaining a relative dynamic balance.’ Thus, it can promote liver fat consumption and transport, and have a certain therapeutic effect on the fatty liver. Xiao et al. (2017) [26] treated NAFLD patients with abdominal massage and found that it can markedly improve the liver function, blood lipids, and liver fibrosis indicators of patients, and its cure rate and effective rate were higher hugely than those of the control group.

Currently, there is certain research on abdominal massage and lactobacillus in the treatment of NAFLD, but there is no report on the combination of the two in the treatment of NAFLD. In this experiment, lactobacillus and abdominal massage were innovatively applied to the rat NAFLD model. The results showed that the FITC-Dextran content of NAFLD rats fed with a high-fat diet elevated greatly (*P* < 0.01), proving that the intestinal permeability of NAFLD rats did increase. After the intervention of lactobacillus, abdominal massage, and combined treatment, the content of FITC-Dextran reduced extremely (*P* < 0.01), proving that treatment can reduce intestinal permeability. The results of HE staining of liver tissue revealed that lactobacillus, abdominal massage, and combined therapy can improve liver cell damage caused by modeling. It was found that the combined therapy of the two can markedly promote the liver function of NAFLD rats, and its effect was better than the two treatment methods alone. Therefore, it was considered that lactobacillus combined with abdominal massage was an effective treatment for NAFLD, which can be further investigated.

## 5. Conclusion

The results of this study preliminarily verified the interventional effect of lactobacillus combined with abdominal massage on the permeability of the intestinal mucosa and further revealed the mechanism of action of abdominal massage in the treatment of NAFLD by improving the permeability of the intestinal mucosal barrier. The results of this study will help to more comprehensively and in-depth reveal the key points and ways of Lactobacillus combined abdominal massage therapy on NAFLD, and provide a scientific basis for the overall improvement of the application level of lactobacillus combined abdominal massage therapy. Although this experiment confirmed that Lactobacillus combined with abdominal massage therapy had a certain intervention effect on NAFLD, the lack of verification data on other related indicators of NAFLD also failed to solve the molecular mechanism problem. With the continuous development of combined traditional Chinese and Western medicine therapy, it is believed that combined traditional Chinese and Western medicine will be the trend in the prevention and treatment of NAFLD in the future. The results of this study preliminarily verified the intervention effect of abdominal massage on intestinal mucosal permeability and further revealed the mechanism of abdominal massage in the treatment of NAFLD by improving intestinal mucosal barrier permeability.

## HIGHLIGHT

1. There was an investigation of the therapeutic effect of Lactobacillus johnsonii combined with abdominal massage on nonalcoholic fatty liver disease (NAFLD).

2. The results of this study can provide a reference for improving the clinical treatment effect of patients with NAFLD.

3. The effect of Lactobacillus johnsonii and abdominal massage on intestinal mucosal barrier permeability was preliminarily verified.
